# Analysis of the Conditions That Affect the Selective Processing of Endogenous Notch1 by ADAM10 and ADAM17

**DOI:** 10.3390/ijms22041846

**Published:** 2021-02-12

**Authors:** Rolake O. Alabi, Jose Lora, Arda B. Celen, Thorsten Maretzky, Carl P. Blobel

**Affiliations:** 1 Tri-Institutional MD/PhD Program, Memorial Sloan-Kettering Cancer Center, Weill Cornell Medicine, Rockefeller University, New York, NY 10021, USA; Rolake.Alabi@ucsf.edu; 2 Arthritis and Tissue Degeneration Program, Hospital for Special Surgery, New York, NY 10021, USA; jol3005@med.cornell.edu (J.L.); arda.celen@nyulangone.org (A.B.C.); 3 Department of Physiology, Biophysics and Systems Biology, Weill Cornell Medicine, New York, NY 10021, USA; 4 Inflammation Program and Department of Internal Medicine, Roy J. and Lucille A. Carver College of Medicine, University of Iowa, Iowa City, IA 52242, USA; thorsten-maretzky@uiowa.edu; 5 Department of Medicine, Weill Cornell Medicine, New York, NY 10021, USA

**Keywords:** ADAM10, ADAM17, Notch1, proteolysis, Notch receptor, Notch pathway, regulation, cell signaling, intercellular signaling, juxtacrine signaling

## Abstract

Notch signaling is critical for controlling a variety of cell fate decisions during metazoan development and homeostasis. This unique, highly conserved signaling pathway relies on cell-to-cell contact, which triggers the proteolytic release of the cytoplasmic domain of the membrane-anchored transcription factor Notch from the membrane. A disintegrin and metalloproteinase (ADAM) proteins are crucial for Notch activation by processing its S2 site. While ADAM10 cleaves Notch1 under physiological, ligand-dependent conditions, ADAM17 mainly cleaves Notch1 under ligand-independent conditions. However, the mechanism(s) that regulate the distinct contributions of these ADAMs in Notch processing remain unclear. Using cell-based assays in mouse embryonic fibroblasts (mEFs) lacking ADAM10 and/or ADAM17, we aimed to clarify what determines the relative contributions of ADAM10 and ADAM17 to ligand-dependent or ligand-independent Notch processing. We found that EDTA-stimulated ADAM17-dependent Notch1 processing is rapid and requires the ADAM17-regulators iRhom1 and iRhom2, whereas the Delta-like 4-induced ligand-dependent Notch1 processing is slower and requires ADAM10. The selectivity of ADAM17 for EDTA-induced Notch1 processing can most likely be explained by a preference for ADAM17 over ADAM10 for the Notch1 cleavage site and by the stronger inhibition of ADAM10 by EDTA. The physiological ADAM10-dependent processing of Notch1 cannot be compensated for by ADAM17 in *Adam10-/-* mEFs, or by other ADAMs shown here to be able to cleave the Notch1 cleavage site, such as ADAMs9, 12, and 19. Collectively, these results provide new insights into the mechanisms underlying the substrate selectivity of ADAM10 and ADAM17 towards Notch1.

## 1. Introduction

The Notch signaling pathway is critical for controlling a variety of cell fate decisions during metazoan development and homeostasis [1,2,3]. It is a unique, highly conserved signaling pathway that relies on cell-to-cell engagement and on the proteolytic release of the intracellular domain of Notch [4,5]. Notch receptors are synthesized in the endoplasmic reticulum and then travel through the secretory pathway to the cell surface. During transport to the cell surface, Notch receptors are processed by furin-type pro-protein convertases in the trans Golgi network [6,7]. This initial, constitutive cleavage at a position referred to as site 1 (S1) gives rise to the mature, functional Notch receptors that exist as heterodimers at the cell surface [6,7].

The initiation of Notch signaling depends on the engagement of a membrane-anchored Notch receptor (i.e., Notch1, 2, 3, or 4 in mammals) to a Notch ligand (i.e., Delta-like (Dll) 1, 3, or 4; Jagged1; or Jagged2) expressed on the cell membrane of a neighboring cell [4,5,8,9]. Following Notch receptor-ligand binding, two sequential cleavage events lead to the release of the Notch intracellular domain (NICD), allowing it to move into the nucleus, where it binds to the transcription factor CSL/RBPJ (CBF-1, Suppressor of Hairless, Lag-2/Recombination signal binding protein for immunoglobulin kappa J region) and regulates the transcription of Notch target genes [10,11]. Genetic studies in *Drosophila* uncovered an important role of a member of the A Disintegrin and Metalloproteinase (ADAM) family of metalloproteases called kuzbanian (also referred to as ADAM10) in the second cleavage event at the site 2 (S2) [12,13,14]. However, initial studies using mammalian cell lines suggested that the related metalloprotease ADAM17, and not ADAM10, was the major protease responsible for the Notch S2 cleavage [15,16]. Finally, foundational work in the field showed that the processing of the Notch1 receptor in its transmembrane domain at a 3rd site (S3), between G1743 and V1744 in mammalian Notch1, by gamma-secretase was required for releasing the NICD from the cell membrane—an essential step in Notch signaling [11,17].

**Figure 1:**
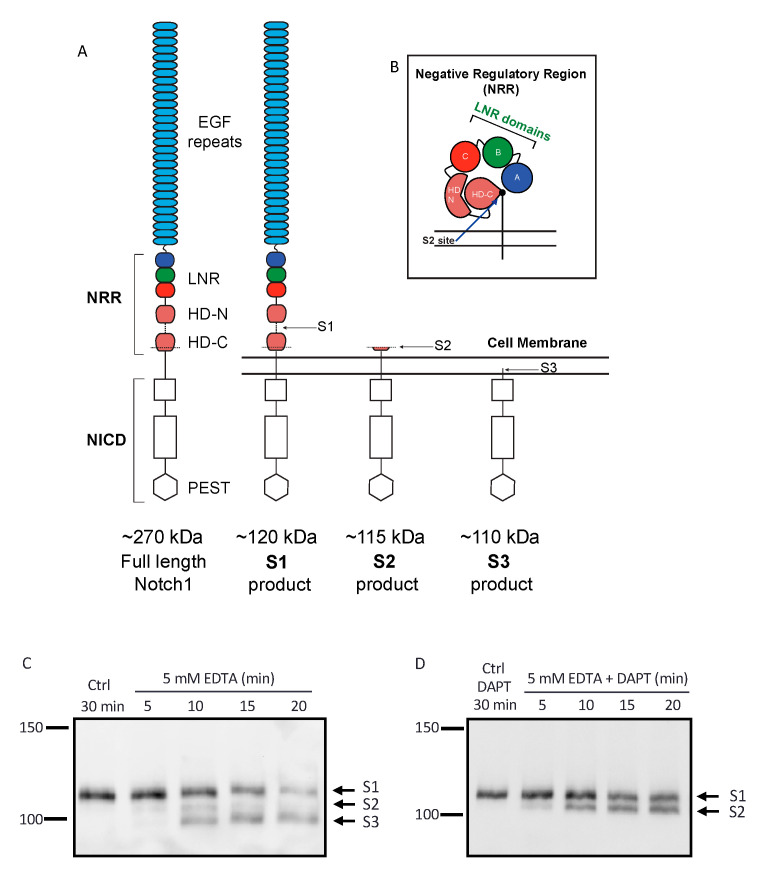
The Notch1 receptor S2 cleavage product is transient and rapidly converted into the S3 cleavage product after treatment with the calcium-chelating agent ethylenediaminetetraacetic acid EDTA. (**A**) Schematic of the Notch1 receptor and (**B**) of its negative regulatory region (NRR, schematic adapted from Gordon et. al, [8,18]). (**C**,**D**) Wild-type mouse embryonic fibroblasts (WT mEFs) were treated with phosphate buffered saline (PBS, vehicle control, Ctrl) for 30 min or with 5 mM EDTA, which activates Notch1 [22] for 5, 10, 15, or 20 min, in the absence (C) or in the presence of 5 µM of gamma-secretase inhibitor *N*-[*N*-(3,5-Difluorophenacetyl)-l-alanyl]-*S*-phenylglycine t-butyl ester( DAPT) (D). The Western blots were probed with an antibody against the cytoplasmic domain of the Notch1 receptor. A fragment migrating close to the expected size of the Notch1 S2 product (~115 kDa) appeared after 10 min of EDTA treatment without DAPT (C) but started to disappear after 15 min of EDTA treatment, suggesting that the S2 product is transient in nature. A more stable and smaller fragment migrating close to the expected size of the S3 product appeared after 10 min of EDTA treatment without DAPT and persisted (C). Note that since the furin-dependent S1 cleavage is a constitutive event, the S1 product is present even in the absence of Notch1 activation with EDTA. (D) When WT mEFs were treated with 5 mM of EDTA in the presence of DAPT for 5, 10, 15, or 20 min, the S2 product began to accumulate after 5 min and appeared to reach maximum accumulation after 10 min. The Western blots shown are representative of at least 3 independent experiments. Abbreviations: EGF, Epidermal Growth Factor; LNR, Lin-12/Notch repeat; HD-N, HD-C, *N*- and *C*-terminal heterodimerization domain; PEST, peptide sequence that is rich in proline (P), glutamic acid (E), serine (S), and threonine (T) residues.

Processing at the S2 site is strictly controlled by a negative regulatory region (NRR) in the juxtamembrane region of Notch receptors [18]. The physiological ADAM-dependent S2 cleavage of Notch1 depends on ligand binding to the extracellular domain of the receptor. When the Notch ligand is endocytosed, this is thought to provide a sufficient mechanical force to unravel the Notch NRR, composed of 3 Lin-12/Notch repeat (LNR) domains and an *N*-terminal and *C*-terminal heterodimerization (HD) domain, thereby uncovering the S2 site for processing (Figure 1) [19,20,21].

In addition to ligand-dependent Notch activation, which is likely responsible for most, if not all, physiological Notch signaling, several modes of Notch activation allow for processing to occur in the absence of ligand binding. One mode of ligand-independent Notch signaling depends on the exposure of the Notch receptor to the chelating agent ethylenediaminetetraacetic acid (EDTA) [22]. EDTA allows access to the Notch S2 site in the absence of ligand binding via the disruption of the protective Notch NRR structure [19]. Moreover, it has been proposed that factors that disrupt hydrogen bonding in the NRR, such as change in ion concentration or pH during endocytosis, could result in the ligand-independent unfolding of the LNR repeats in the NRR of Notch [18,23,24,25]. Notably, gain-of-function mutations in Notch that are found in more than 50% of cases of childhood cancer T-cell acute lymphoblastic leukemia (T-ALL) result from point mutations in the Notch1 NRR, which cause the destabilization of the NRR and result in constitutive access to the Notch S2 site and Notch activation [24,26,27].

Even though both ADAM10 and ADAM17 have been implicated in the S2 processing of Notch, results from genetic studies in multiple species (including *C. elegans*, *Drosophila,* and mice) have shown that ADAM10 is the enzyme that is responsible for physiological Notch signaling during development in vivo [12,13,16,28,29,30,31,32,33]. While ADAM10-null mice closely resemble Notch1-null mice (including exhibiting embryonic lethality at E9.5 and defects in somitogenesis), ADAM17 null mice resemble mice lacking the epidermal growth factor receptor (EGFR) or EGFR ligands such as transforming growth factor α (TGFα) [30,34,35]. In addition, several ADAM10 conditional knockout mice (including mice with specific inactivation in neurons, T-cells, keratinocytes, and endothelial cells) have been reported to exhibit Notch-related phenotypes [31,32,36,37,38,39]. Moreover, cell-based assays tracking processing at the S2 site of full-length overexpressed Notch1 have identified ADAM10, and not ADAM17, as the major protease responsible for cleaving Notch1 in ligand-dependent Notch1 signaling [28,40]. In contrast, ADAM17 has typically been found to only be able to cleave Notch1 under non-physiological conditions, such as following treatment with EDTA or in the presence of disruptive NRR mutations [28,40,41]. Thus, while in vitro studies using cell-based assays suggest that ADAM17 cleaves the Notch1 receptor, the overall body of in vivo evidence overwhelmingly supports a role for ADAM10 as the primary physiological sheddase of Notch1. Here, we established cell-based assays to analyze endogenous Notch1 processing by ADAM10 or ADAM17 to elucidate the mechanisms that control the different contributions of these two ADAMs to the EDTA- versus ligand-induced processing of Notch1.

## 2. Results

### 2.1. Establishing an Assay to Evaluate Endogenous Notch1 Processing by ADAM10 and ADAM17

The first goal of this study was to establish a Western blotting protocol that would allow us to monitor the processing of endogenous Notch1 receptor by endogenous ADAMs and to simultaneously visualize the S1, S2, and S3 cleavage products of the Notch1 receptor (Figure 1A,B). We found that a monoclonal antibody to the Notch1 cytoplasmic domain that recognized the membrane-anchored 120 kDa subunit of Notch1 on Western blots of wild-type (WT) mouse embryonic fibroblasts (mEFs) was suitable for this purpose. Specifically, when WT mEFs were treated with EDTA, which unravels the Notch1 NRR to expose the Notch1 S2 site [22,42], we observed the appearance of products with molecular weights corresponding to the sizes expected for the S2 and S3 products after 10 min (Figure 1C, 115 kDa for S2, 110 kDa for S3). The putative S2 product present after 10 min of EDTA treatment started to disappear after 15 min of EDTA treatment. In agreement with these findings, when cells were treated with EDTA in the presence of gamma-secretase inhibitor DAPT, the levels of the Notch1 S2 product stabilized after about 10 min (Figure 1D).

### 2.2. Endogenous ADAM17 Cleaves at the EDTA-Exposed Notch1 S2 Site

**Figure 2:**
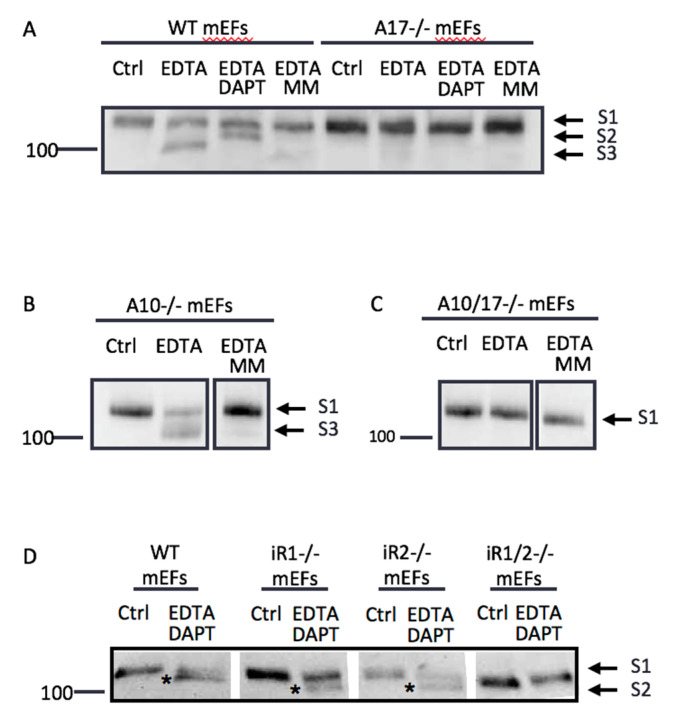
ADAM17 is the primary S2 protease involved in EDTA-induced endogenous Notch1 processing. Cells were treated with PBS (vehicle control, Ctrl), 5 mM of EDTA, or 5 mM of EDTA in the presence of the inhibitors DAPT (5 µM) or marimastat (5 µM MM, metalloprotease inhibitor that blocks ADAMs10 and 17) for 15 min. MM blocks the S2 cleavage, whereas DAPT blocks S3 processing. All the inhibitors were pre-incubated for 2 h in Opti-MEM and Western blots were performed using an anti-Notch1 cytoplasmic domain antibody. (**A**) Samples of WT mEFs treated with 5 mM of EDTA showed a band around the expected size of S3, which was not produced in the presence of DAPT. Instead, a fragment around the expected size of the Notch1 S2 product accumulated upon DAPT treatment. WT samples treated with EDTA and MM showed no S2 and S3 products. In contrast, mEFs lacking ADAM17 (*A17-/-* mEFs) showed no S2 or S3 products under the conditions where these bands were observed for WT mEFs. (**B**) ADAM10-deficient mEFs (*A10-/-* mEFs) showed the accumulation of an S3 product after EDTA treatment, which was blocked by MM, just like in WT mEFs. (**C**) Double-knockout mEFs deficient in ADAM10 and ADAM17 (*A10/17-/-* mEFs) resembled *A17-/-* mEFs in that they showed no S3 production in response to EDTA treatment. (**D**) mEFs deficient in the ADAM17 regulators iRhoms 1 and 2 *(iR1/2-/-* mEFs) also produced no S2 fragment in the presence of EDTA and DAPT, whereas the inactivation of either iRhom1 or iRhom2 (*iR1-/-* or *iR2-/-* mEFs) did not prevent the generation of Notch1 S2 (indicated by an asterisk). All Western blots shown are representative of at least 3 independent experiments.

To determine if the production of this EDTA-induced S2 product is dependent on ADAM10, ADAM17, or both, we performed similar experiments in mEFs deficient in *Adam10* (*A10-/-*), *Adam17* (*A17-/-*), or both (*A10/17-/-*). When WT mEFs were treated with EDTA, a fragment at the expected size of the S3 product accumulated (Figure 2A, see also Figure 1C). The production of the S3 band in EDTA-treated WT mEFs was blocked by the gamma-secretase inhibitor DAPT, which promoted the accumulation of the S2 product. The appearance of the S2 and S3 products was completely prevented by treatment with the metalloprotease inhibitor marimastat (MM, Figure 2A). In contrast, *A17-/-* mEFs produced no S3 fragment in the presence of EDTA. Interestingly, *A10-/-* mEFs still yielded S3 product in response to EDTA treatment (Figure 2B), while *A10/17-/-* mEFs produced no S3 band following the addition of EDTA (Figure 2C). Similar to *A17-/-* mEFs, mEFs lacking both recently discovered regulators of ADAM17, the inactive Rhomboids (*iRhoms*) 1 and 2, did not produce the Notch1 S2 fragment after the addition of EDTA in the presence of DAPT (Figure 2D), whereas mEFs lacking either *iRhom1* or *iRhom2* were able to generate the Notch1 S2 fragment under these conditions. These results corroborate that ADAM17, controlled by its regulators iRhom1 or iRhom2, but not ADAM10, is the major protease responsible for endogenous Notch1 processing under non-physiological conditions in the presence of EDTA.

### 2.3. ADAM10 Cleaves at the S2 Site in Endogenous Notch1 Exposed by Culture of Cells on Immobilized Dll4

**Figure 3:**
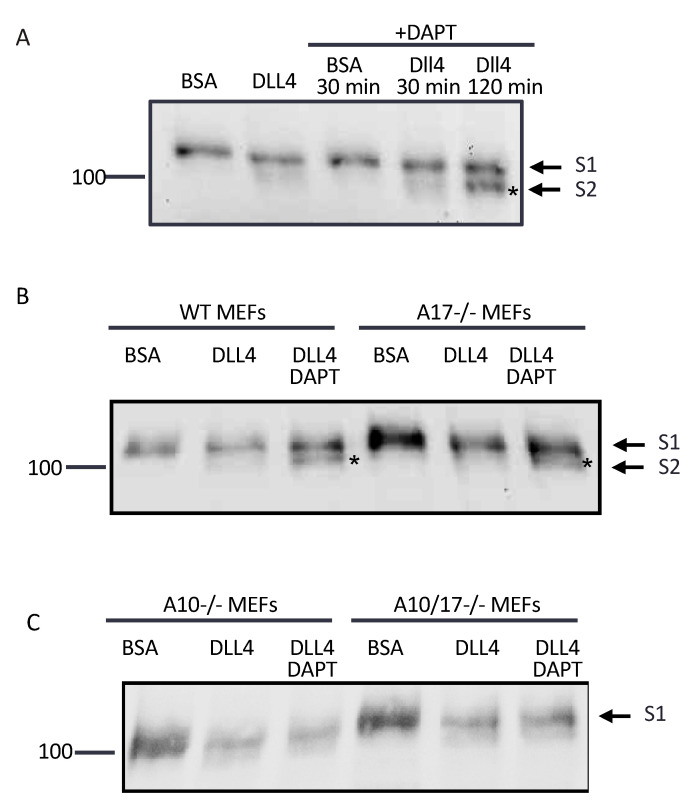
ADAM10, and not ADAM17, cleaves the S2 site in Dll4 ligand-induced endogenous Notch1 processing. Cells were plated on tissue culture dishes coated with 1 µg/mL of the Notch1 ligand Dll4 in bovine serum albumin (BSA) or with BSA alone. Cells were starved in Opti-MEM for 2 h prior to treatment with DAPT or incubated with Opti-MEM alone for an additional 30 min or 120 min. (**A**) WT mEFs plated on Dll4 alone showed the minimal accumulation of an S2 product. Treatment with DAPT for 30 min resulted in very little accumulation of S2 compared to the accumulation after 120 min of DAPT treatment, when the S2 accumulation was substantial. (**B**) In WT mEFs, S2 product accumulated in cells plated on Dll4 that were treated with DAPT for 2 h. A similar accumulation of S2 product in DAPT-treated cells was apparent in *A17-/-* mEFs plated on Dll4. (**C**) In contrast, *A10-/-* mEFs, similar to *A10/17-/-* mEFs, plated on Dll4 showed no accumulation of S2 in the presence of DAPT. The Western blots are representative of at least 3 independent experiments.

We next determined which ADAM is responsible for endogenous Notch1 cleavage when mEFs were cultured on tissue culture plates coated with the Notch1 ligand Dll4, which more closely resembles a physiologically relevant situation. WT mEFs plated on bovine serum albumin (BSA) as a control showed only the S1 band by Western blot, whereas a hint of an S2-like band was present when WT mEFs were plated on Dll4-coated plates after 2 h. Unlike in EDTA-treated samples, no S3 band was visible after plating WT mEFs on Dll4, presumably because Dll4-induced processing is slower and less efficient compared to EDTA-induced processing, so that the S3 band does not accumulate at detectable levels. When WT mEFs plated on Dll4 were treated with DAPT, which leads to an accumulation of S2 by blocking gamma secretase upon EDTA treatment (Figure 1D), this yielded a faint S2-like band after 30 min and a clear S2-like product after 2 h (Figure 3A). When *A17-/-* mEFs were plated on Dll4 and treated with DAPT for 2 h, the Notch1 S2-like product accumulated to similar levels as in WT mEFs under these conditions (Figure 3B). However, when *A10-/-* and *A10/17-/-* mEFs were plated on Dll4 and treated with DAPT for 2 h, a faint S2-like band was visible in the presence or absence of DAPT, but unlike in WT and *A17-/-* mEFs, there was no evident accumulation of this S2-like band in DAPT-treated samples compared to untreated controls (Figure 3C). We noted that the levels of the S1 band decreased upon plating on Dll4, especially in *A10-/-* and *A10/17-/-* mEFs, presumably because plating on Dll4 triggers the endocytosis of Notch receptors, whereas plating on bovine serum albumin (BSA)-coated tissue culture dishes does not. These results support the conclusion that ADAM10, but not ADAM17, is responsible for cleaving the Notch1 S2 site in a manner that promotes gamma-secretase-dependent S3 production during the ligand-induced processing of endogenous Notch1 on Dll4-coated plates.

### 2.4. The Notch1 S2 Site Is Protected from Processing by Stimulated ADAMs

**Figure 4:**
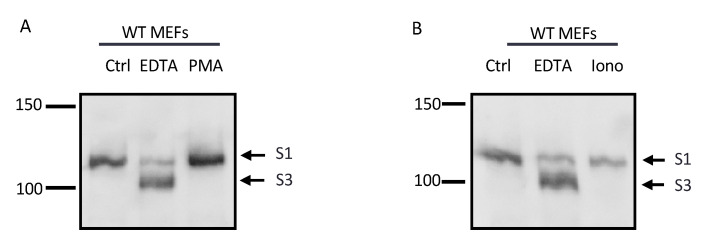
Processing of the endogenous S2 site cannot be induced by the stimulation of ADAM10 or ADAM17. (**A**) WT mEFs were treated with 25 ng/mL of PMA, a strong activator of ADAM17, or (**B**) with 2.5 µM ionomycin, which strongly activates both ADAM10 and 17, with 5 mM EDTA treatment for 30 min included as a positive control for the processing of Notch1. The Western blots shown are representative of at least 3 independent experiments.

Studies of the NRR of Notch receptors suggest that the S2 site is normally well-protected in its native conformation [18,24]. To determine whether the stimulation of ADAM activity is sufficient to, in principle, overcome this barrier to constitutive access by the ADAMs to the Notch1 S2 site, we treated WT mEFs with strong activators of ADAM17 or ADAM10. WT mEFs treated with the phorbol ester phorbol myristate-12-acetate (PMA), which strongly stimulates ADAM17 [43,44,45], or with the ionophore ionomycin, which strongly activates both ADAM10 and ADAM17 [46,47,48,49,50,51], did not yield detectable levels of S2 product, with EDTA stimulation serving as a positive control (Figure 4). These results demonstrate that the stimulation of ADAM10 and ADAM17 is not sufficient to induce processing at the endogenous Notch1 S2 site, so long as this site is protected by the NRR domain.

### 2.5. The Constitutively Exposed S2 Site Behaves Like an ADAM17 Substrate

**Figure 5:**
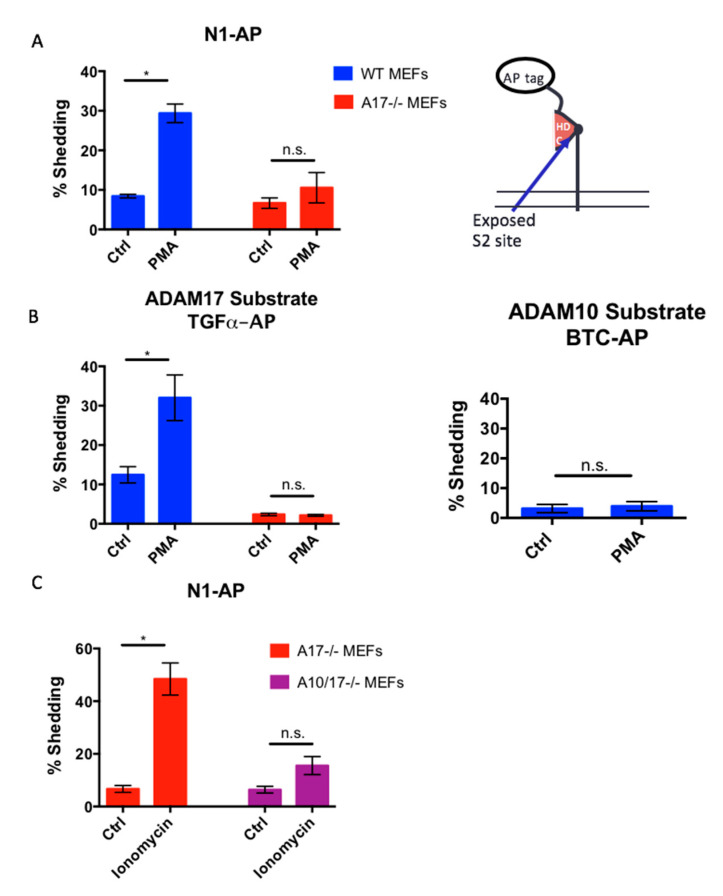
The constitutively exposed Notch1 S2 site behaves like an ADAM17 substrate. (**A**) WT and *A17-/-* mEFs were transfected with a truncated Notch1 receptor, consisting of the *C*-terminal portion of the receptor starting from amino acid residue 1687 within the *C*-terminal part of the heterodimerization domain, with an alkaline phosphatase tag attached to its *N*-terminus (Notch1-AP or N1-AP), and were stimulated with the ADAM17-selective stimulus PMA [52]. WT mEFs stimulated with 25 ng/mL of PMA had increased shedding of N1-AP over untreated WT mEFs. There was no significant increase in the PMA-induced shedding of N1-AP in *A17-/-* mEFs. (**B**) N1-AP shedding thus behaved similarly to the shedding of TGFα-AP, a known ADAM17 substrate [35,52], which could be stimulated by PMA in WT mEFs, but not in *A17-/-* mEFs. In contrast, PMA treatment did not result in the shedding of an established ADAM10 substrate, betacellulin-AP (BTC-AP) [52]. (**C**) In *A17-/-* mEFs treated with 2.5 µM of ionomycin, which strongly stimulates ADAM10 and ADAM17, N1-AP shedding was increased over unstimulated mEFs, whereas there was no statistically significant increase in N1-AP shedding in *A10/17-/-* mEFs treated with ionomycin, suggesting that ADAM10 can shed N1-AP in the absence of ADAM17. All the data are shown as mean ± standard error of the mean (SEM) for *n* ≥ 3 independent experiments. An * indicates *p* < 0.05 and n.s. indicates no statistically significant difference using the unpaired two-tailed Student’s *t*-test.

In order to determine whether the S2 cleavage site is a preferred site for ADAM10 or ADAM17 when it is constitutively exposed, we generated a truncated Notch1 receptor lacking the NRR domain with an alkaline-phosphatase (AP) tag attached *N*-terminally to the exposed Notch1 cleavage site (N1-AP, Figure 5A). This allowed us to compare the properties of N1-AP to a typical ADAM17-selective substrate (transforming growth factor α, TGF α-AP) or a typical ADAM10-selective substrate (betacellulin, BTC-AP) [52]. We found that, in WT mEFs, the shedding of N1-AP was increased in the presence of PMA (25 ng/mL PMA for 1 h), whereas the PMA-stimulated shedding of N1-AP was was not significantly increased in *A17-/-* mEFs (Figure 5A). Under the conditions used here, stimulation with PMA specifically activates the shedding of ADAM17 substrates, such as TGFα-AP, but not of ADAM10 substrates, such as BTC-AP in WT mEFs (Figure 5B) [52]. Moreover, we found that the treatment of *A17-/-* mEFs with the ADAM10 and ADAM17 stimulant ionomycin [48] also resulted in significantly increased N1-AP shedding, which was strongly reduced in *A10/17-/-* mEFs (Figure 5C). These results suggest that ADAM17 can efficiently cleave the constitutively exposed Notch1 S2 site, which behaves like an ADAM17-selective cleavage site, although ADAM10 can also cleave the exposed S2 site when it is stimulated with ionomycin in the absence of ADAM17.

### 2.6. Differential Effects of EDTA on ADAM10 and ADAM17 Activity

**Figure 6:**
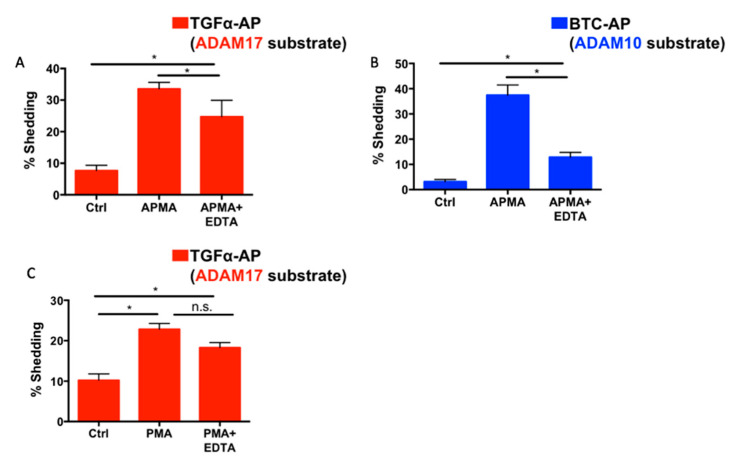
ADAM10 activity is more strongly inhibited by EDTA than ADAM17. (**A**,**B**) After 6 h of starvation in Opti-MEM, WT mEFs were treated with 4-amino-phenylmercuric acetate (APMA, 200 µM) in the presence or absence of 5 mM of EDTA for 30 min. APMA treatment induced the shedding of the ADAM17 substrate TGFα-AP (A) and the ADAM10 substrate BTC-AP (B). APMA-induced BTC-AP shedding, which depends on ADAM10, was more strongly reduced by treatment with 5 mM of EDTA than APMA-induced TGFα-AP shedding, which depends on ADAM17. In addition, PMA-induced TGFα-AP shedding, which depends on ADAM17, was not significantly reduced in the presence of EDTA (**C**). Data are shown as mean ± SEM for *n* ≥ 3 independent experiments. * indicates *p* < 0.05 and n.s. indicates no statistically significant difference using one-way ANOVA followed by Tukey’s multiple comparisons test.

As we and other groups have shown, Notch1 is preferentially cleaved by ADAM17 in the presence of EDTA [28,40]. This finding is unexpected given the abundant genetic evidence that ADAM10 is the physiologically relevant processing enzyme for Notch1 [13,14,29,30,31,32,37,38]. To determine if differences in each ADAM’s activity status might influence the contribution of ADAMs 10 and 17 to Notch1 processing, we tracked changes in ADAM10 and ADAM17 activity in the presence of 5 mM of EDTA using selective AP shedding assays. For these experiments, we chose the dual ADAM10/ADAM17 stimulus 4-amino-phenylmercuric acetate (APMA) [46,53], which does not depend on calcium-influx and therefore provided an activation mechanism that should not be directly blocked by EDTA. WT mEFs treated with APMA exhibited the increased shedding of both the ADAM10-selective substrate BTC-AP and the ADAM17-selective substrate TGFα-AP (Figure 6A). Notably, in the presence of EDTA the APMA-induced shedding of the ADAM10 substrate BTC-AP was more significantly reduced than the APMA-induced shedding of the ADAM17-dependent TGFα-AP (Figure 6B). In addition, PMA-induced TGFα-AP shedding was also not significantly inhibited by EDTA (Figure 6C). Taken together, these data suggest that ADAM10 activity is more effectively inhibited by EDTA than ADAM17 activity, providing a possible explanation for why ADAM17 is the main Notch1 processing enzyme in EDTA-treated cells.

### 2.7. Other ADAMs Can Cleave the Exposed Notch1 S2 Site

**Figure 7:**
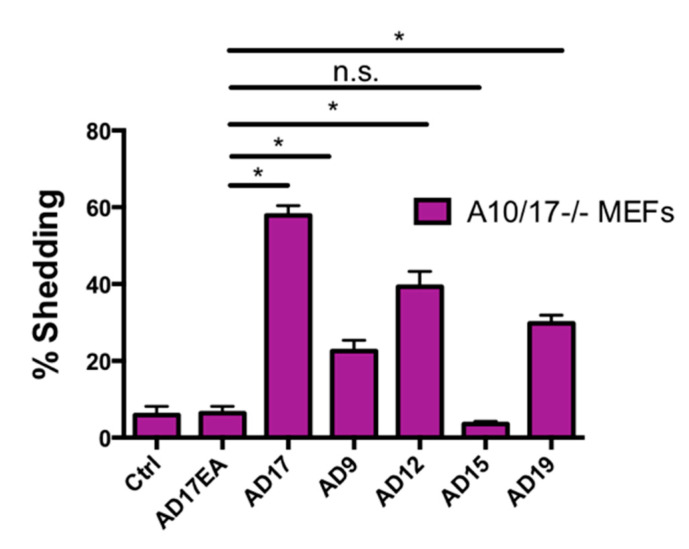
ADAMs 9, 12, and 19 can cleave N1-AP in *A10/17-/-* mEFs. Overexpression of ADAMs 9, 12, 15, 17, and 19 in *A10/17-/-* mEFs shows that all but ADAM15 can increase the processing of N1-AP compared to the catalytically inactive ADAM17E>A control. Data are shown as mean ± SEM for *n* ≥ 3 independent experiments (*n* = 3 for ADAM17E>A mutant, *n* = 6 for all other samples). * indicates *p* < 0.05 using the unpaired 2-tailed Student’s *t*-test. The difference between the control sample, in which only N1-AP was expressed, and the sample in which the inactive ADAM17E>A was co-expressed with N1-AP was not statistically significant (not indicated on the figure), and the difference between N1-AP shedding in the presence of overexpressed inactive ADAM17E>A and ADAM15 also did not reach statistical significance (n.s.).

Since both ADAM10 and ADAM17 can cleave Notch1, albeit under different conditions, we used N1-AP with an exposed S2 site to determine whether other ADAMs can, in principle, process Notch1 when they are overexpressed in *A10/17-/-* mEFs. As a positive control, we overexpressed ADAM17, which could promote constitutive N1-AP shedding, whereas the inactive ADAM17E>A mutant did not (Figure 7). Under these conditions, the overexpression of ADAM9, ADAM12, and ADAM19, but not ADAM15, increased the shedding of N1-AP compared to overexpression of the inactive ADAM17E>A (Figure 7). These results suggest that the exposed N1 cleavage site can, in principle, also be cleaved by these other ADAMs, at least when they are overexpressed.

## 3. Discussion

The essential role of proteolysis in activating Notch receptors has generated a substantial amount of interest in understanding the underlying mechanism and the responsible proteases. While genetic studies clearly implicate ADAM10 [13,14,29,30,31,32,37,38,54], a survey of the literature shows that numerous studies consider ADAM17 to be the primary Notch1 processing enzyme [15,55,56,57,58,59,60,61,62]. Previous studies with overexpressed Notch1 support the genetic studies by showing that ligand-induced Notch1 processing depends on ADAM10 [28,40]. With the ability to track the specific cleavage products during endogenous Notch1 receptor processing, we were able to corroborate the essential contribution of ADAM10, but not ADAM17, to the ligand-induced processing of endogenous Notch1 in mEFs plated on Dll4. We could also show that the EDTA-induced activation of endogenous Notch1 depends on ADAM17, and not ADAM10. This is the first biochemical evidence, to our knowledge, that the stimulation of Notch1 processing by Dll4, a key regulator of Notch signaling in endothelial cells, depends on endogenous ADAM10. This dependence of ligand-induced Notch1 cleavage on ADAM10 is in agreement with previous studies using the Notch ligands Dll1 and Jag1 [28].

Interestingly, in contrast to the prolonged time required for the accumulation of the Dll4-dependent S2 product, EDTA-induced Notch1 processing led to the rapid accumulation of the Notch1 S2 (within 10 min). This suggests that EDTA is a more efficient inducer of the Notch1 NRR unraveling process than Dll4 binding. Although EDTA remains a commonly used inducer of Notch signaling in the field, it seems possible that both the speed and strength of EDTA-induced Notch1 signaling activity do not correlate well with the more physiologically relevant Dll4-induced or other ligand-induced Notch signals.

Along with its accelerated activation of Notch1 signaling, EDTA is also unusual due to its dependence on ADAM17, and not ADAM10, for S2 cleavage. Given the literature that supports the essential role of ADAM10 in Notch signaling in vivo, the dependence of EDTA-induced Notch cleavage on ADAM17 is unexpected. Using a truncated Notch1 receptor with an exposed S2 site, we found that the Notch1 S2 cleavage site resembles a typical ADAM17 substrate and not an ADAM10 substrate in its activity profile. This suggests that the Notch1 S2 site (alanine-1710/valine-1711) is a preferred ADAM17 cleavage site. This finding is further supported by other work that indicates that ADAM17 mainly prefers valine in the P1′ position (a valine is found in the P1′ of the Notch1 S2 cleavage site), while ADAM10 mainly prefers leucine and aromatic residues in the P1′ position [63,64].

In addition to this contribution of cleavage site specificity, our finding that the Notch activator EDTA inhibits ADAM10 activity more strongly than ADAM17 activity likely accounts for the unusual dependence of EDTA-dependent Notch1 cleavage on ADAM17. This result is supported by biochemical studies in the literature that show that while ADAM17 activity is poorly inhibited by EDTA, ADAM10 activity is more sensitive to inhibition by EDTA [65,66,67].

Our previous findings in conditional knockout mice lacking ADAM10 and ADAM17 in endothelial cells [32] suggest that, while in principle ADAM17 can efficiently cleave the exposed Notch1 S2 site, it does not do so under physiological ligand-induced conditions—even in the absence of ADAM10. This observation seems to indicate that other mechanisms must exist that prevent ADAM17 cleavage of the ligand-dependent exposed Notch1 S2 site.

One potential explanation for this could be that the Notch1 receptor exists in complexes in the cell membrane so that it is available for processing by ADAM10 but inaccessible to ADAM17. Evidence in the literature suggests that the TspanC8 family of tetraspanins, which have been shown to be required for the proper maturation and trafficking of ADAM10, might be candidate ADAM10-Notch1 complex partners [68,69,70,71]. In fact, work conducted in *Drosophila* suggests that silencing TspanC8 family members results in decreased Notch activity [69]. In addition, there is evidence that ADAM10 also exists in complex with gamma-secretase [72]. In contrast to ADAM10’s dependence on TspanC8 tetraspanins, a different class of membrane proteins referred to as inactive Rhomboids (iRhoms) is required for the maturation and function of ADAM17 [73,74,75]. Our results with iRhom-deficient cells showed that iRhom1 and iRhom2 can both support ADAM17-dependent Notch1 processing upon EDTA stimulation. However, the inactivation of both iRhoms, which prevents the maturation and functional activation of ADAM17, abolishes EDTA-stimulated Notch1 processing, just like the inactivation of ADAM17. Further work must be conducted to determine if ADAM10 and tetraspanins do indeed exist in complexes with Notch1 receptor under physiological conditions.

We hope that our efforts to better understand the mechanisms that regulate the contributions of ADAM10 and ADAM17 to the processing of endogenous Notch1 will help to resolve inconsistencies in the literature concerning the relevant Notch1 S2 protease. The early studies that identified ADAM17 and not ADAM10 as the relevant metalloprotease in Notch1 processing relied on truncated Notch1 constructs to assay S2 cleavage or on biochemical studies to purify the Notch1 processing enzyme from cell lysates [15,16]. Our data showing that the exposed S2 site is a preferred ADAM17 cleavage site provides a plausible explanation for this finding. On the other hand, the genetic and cell biological studies that point to ADAM10 as the relevant protease in ligand-dependent Notch signaling could be explained by a growing literature that suggests that ADAM10, gamma-secretase, tetraspanins, and perhaps even Notch may be found together in membrane complexes [69,70,71,72]. It is also possible that the conformational change of the NRR induced by ligand binding is smaller than following treatment with EDTA, which could conceivably only provide access to the S2 cleavage site to ADAM10, but not to ADAM17.

Recent findings about the differential regulation of ADAMs 10 and 17 suggest that further study of the individual contributions of ADAM10 and ADAM17 to Notch signaling in different contexts promise to improve our understanding of the physiological regulation of this crucial signaling pathway. Notably, there is some evidence that ADAM17 may cleave Notch in at least one case of ligand-independent Notch activation that occurs in vivo [41]. Specifically, the immature thymocytes of T-cell acute lymphoblastic leukemia (T-ALL) express mutant forms of Notch1 receptors with destabilized NRRs [26,27,76]. The results of one study suggest that the Notch1 S2 cleavage in these T-ALL mutants is dependent on both ADAM10 and ADAM17 [41]. Since, as previously noted, the maturation and activity of ADAM10 and ADAM17 are regulated by distinct classes of membrane proteins (Tspan C8 and iRhoms, respectively), these regulatory binding partners of ADAM10 and ADAM17 may emerge—in addition to the ADAMs they bind to—as attractive targets in the treatment of T-ALL.

Future work on ADAM17/iRhom-dependent Notch signaling in T-ALL may prove especially fruitful. Previous studies completed in our laboratory suggest that the deletion of iRhom2 is sufficient to eliminate or severely limit ADAM17 maturation in immune cells [77]. Due to partial redundancy with iRhom1 in other cell types [75], targeting iRhom2 alone would leave ADAM17 function largely intact in other non-immune cell types—thus avoiding the potential side effects of pan-ADAM17 inhibition. This makes an iRhom2 targeting strategy a more specific approach to inhibiting ADAM17-dependent (ligand-independent) Notch signaling, and also opens a potentially translationally relevant new avenue in investigation of T-ALL treatment. Collectively, we hope that the work presented here will contribute to clarifying the importance of ADAM10 and ADAM17 function in ligand-dependent and ligand-independent Notch signaling.

## 4. Materials and Methods

### 4.1. Cell Lines, Reagents and Antibodies

Embryonic fibroblasts from *Adam10-/-*, *Adam17-/-*, *Adam10/17-/-*, *iRhom1-/-*, *iRhom2-/-*, and *iRhom1/2-/-* have been previously described [48,75]. *N*-[*N*-(3,5-Difluorophenacetyl)-l-alanyl]-*S*-phenylglycine t-butyl ester (DAPT) was obtained from Sigma-Aldrich (St. Louis, MO, USA). Phorbol-12 myristate 13-acetate (PMA), 4-aminophenylmercuric acetate (APMA), and ethylenediaminetetraacetic acid (EDTA) were from Sigma-Aldrich (St. Louis, MO, USA). Ionomycin was from Calbiochem. Marimastat was a gift from Ouathek Ouerfelli (Sloan-Kettering Institute, New York, NY, USA) [78]. The anti-Notch1 (cytoplasmic) antibody (clone EP1238Y) was from Millipore (Temecula, CA, USA). The HRP-conjugated anti-rabbit secondary antibody was from Promega (Madison, WI, USA). Recombinant mouse Delta-like 4 (Dll4) protein (Ser28-Pro525 with a *C*-terminal 10-His tag, catalog number 1389-D4) was from R&D Systems (Minneapolis, MN, USA).

### 4.2. Plasmids

Alkaline phosphatase tagged TGFα (TGFα-AP) and betacellulin (BTC-AP) have been previously described [47,48,52]. To generate the Notch1-AP construct with the constitutively exposed S2 site, a truncated fragment of mouse Notch1 (with amino acid 1687 at its *N*-terminal end) was cloned into the pAPtag5 vector (GenHunter, Nashville, TN, USA). Expression vectors for WT mouse ADAM17, the catalytically inactive ADAM17 E→A(A17E/A), WT mouse ADAM9, ADAM12, ADAM15, and ADAM19 have been described previously [52,79,80]

### 4.3. Cell Transfection and Alkaline Phosphatase (AP) Ectodomain Shedding Assay

Mouse embryonic fibroblasts were transfected with the relevant plasmids using Genjet (SignaGen, Ijamsville, MD, USA) or Lipofectamine 2000 (Life Technologies, Carlsbad, CA, USA) with comparable results. One day following transfection, cells were incubated in Opti-MEM medium for at least 2 h prior to the stimuli treatment. Cells were treated with stimuli (2.5 µM ionomycin, 25 ng/mL PMA, or 200 µM APMA) in Opti-MEM for 45 min. AP activity in supernatants and lysates was measured by colorimetry as previously described [81].

### 4.4. Western Blot Analysis

Cells were lysed in 1% Triton-X or RIPA buffer (1% Triton-X, 0.5% sodium deoxycholate, 50 mM Tris HCl, 150 mM NaCl, 0.1% SDS, 2 mM EDTA). Samples were separated on SDS-polyacrylamide gels (6% gels for anti-Notch1 blots) and transferred to nitrocellulose membranes (Pall Life Science, Port Washington, NY, USA) by semi-dry transfer (at 105 mAmps per gel, for 60 to 90 min). Following blocking (in 5% milk in Tris buffered Saline (TBS) + 0.05% Tween (TBST) for 1 h at room temperature), membranes were incubated overnight at 4 °C with Notch1 antibody (EP1238Y, MilliporeSigma, Burlington, MA, USA, at 1:1000 dilution in 5% milk in TBST). Following 1 rinse and 4 washes in TBST (three 15 min washes and one 5 min wash), the membranes were incubated in horseradish peroxidase (HRP)-coupled secondary antibody dissolved in 5% milk in TBST (1:2500 secondary antibody dilution for Notch1 blots) for 1 h at room temperature. Blots were washed in TBST (one quick rinse, three 15 min washes, and one 5 min wash) and bands on the membranes were visualized with ChemiDoc (BioRad, Hercules, CA, USA) following incubation with chemiluminescence substrate.

### 4.5. Statistical Analysis

The unpaired 2-tailed Student’s *t*-test was used unless otherwise noted. The Prism 6 (Graph Pad, San Diego, CA, USA) software was used to perform all statistical tests. A *p*-value of < 0.05 was considered statistically significant.
